# Pneumonia and Exposure to Household Air Pollution in Children Under the Age of 5 Years in Rural Malawi: Findings From the Cooking and Pneumonia Study

**DOI:** 10.1016/j.chest.2020.03.064

**Published:** 2020-04-18

**Authors:** Kevin Mortimer, Maia Lesosky, Sean Semple, Jullita Malava, Cynthia Katundu, Amelia Crampin, Duolao Wang, William Weston, Dan Pope, Deborah Havens, Stephen B. Gordon, John Balmes

**Affiliations:** a Liverpool School of Tropical Medicine, Liverpool, UK; b University of Cape Town, Cape Town, South Africa; c Stirling University, Stirling, UK; d Malawi Epidemiology and Intervention Research Unit, Chilumba, Malawi; e London School of Hygiene and Tropical Medicine, London, UK; f University of Liverpool, Liverpool, UK; g Malawi Liverpool Wellcome Trust Programme, Blantyre, Malawi; h University of California, Berkeley, CA; i University of California, San Francisco, San Francisco, CA

**Keywords:** cookstove, household air pollution, pneumonia, CAPS, Cooking and Pneumonia Study, CO, carbon monoxide, COHb, carboxyhemoglobin, IMCI, Integrated Management of Childhood Illness, IQR, interquartile range, IRR, incident rate ratios, LOD, limit of detection, ppm, parts per million, WHO, World Health Organization

## Abstract

**Background:**

Exposure to household air pollution is associated with an increased risk of pneumonia in children in low- and middle-income countries; however, exposure-response data are limited, and there are uncertainties around the extent to which biomass-fueled cookstoves can reduce these exposures.

**Research Question:**

What is the association between exposure to household air pollution and pneumonia in children under the age of 5 years in rural Malawi and what are the effects of a biomass-fueled cookstove intervention on personal exposure to household air pollution?

**Study Design and Methods:**

We measured personal exposure to carbon monoxide (CO; 48 hours of continuous measurement and transcutaneous carboxyhemoglobin) every 6 months in children who participated in a cluster-randomized controlled trial of a cleaner burning biomass-fueled cookstove intervention to prevent pneumonia in children under the age of 5 years in rural Malawi (the Cooking And Pneumonia Study). Exposure-response and multivariable analyses were done.

**Results:**

We recruited 1805 (928 intervention; 877 control) children (mean age, 25.6 months; 50.6% female). We found no evidence of an association between exposure to CO (incident rate ratio, 1.0; 95% CI, 0.967 to 1.014; *P* = .53) or carboxyhemoglobin (incident rate ratio, 1.00; 95% CI, 0.993 to 1.003; *P* = .41) in children who experienced pneumonia vs those who did not. Median exposure to CO in the intervention and control groups was was 0.34 (interquartile range, 0.15 to 0.81) and 0.37 parts per million (interquartile range, 0.15 toa 0.97), respectively. The group difference in means was 0.46 (95% CI, −0.95 to 0.012; *P* = .06).

**Interpretation:**

Exposure to CO in our population was low with no association seen between exposure to CO and pneumonia incidence and no effect of the Cooking And Pneumonia Study intervention on these exposures. These findings suggest that CO may not be an appropriate measure of household air pollution exposure in settings such as rural Malawi and that there is a need to develop ways to measure particulate matter exposures directly in young children instead.

**Clinical Trial Registration:**

ISRCTN59448623.

Malawi has one of the world’s highest infant and <5-year-old children mortality rates (42 and 63 per 1000 live births, respectively, in 2015 to 2016) despite having made progress towards meeting the Millennium Development Goal of reducing child mortality rates.^1^ Pneumonia is the leading cause of death and one of the most common causes of morbidity.^2^^,^^3^

**Take-home Point**

**Study question:** What is the association between exposure to household air pollution and pneumonia in children under the age of 5 years in rural Malawi, and what are the effects of a biomass-fueled cookstove intervention on personal exposure to household air pollution?

**Results:** Exposure to carbon monoxide in our population was low with no association seen between exposure to carbon monoxide and pneumonia incidence and no effect of a biomass-fueled cookstove intervention on these exposures.

**Interpretation:** Carbon monoxide may not be an appropriate measure of household air pollution exposure in settings like rural Malawi. The role of cleaner-burning cookstoves and fuels as standalone health interventions needs to be reexamined.

Exposure to smoke produced when biomass fuels (animal or plant material) are burned in open fires is understood to be a major avoidable risk factor for pneumonia in young children.^4^, ^5^, ^6^ In Africa, biomass fuels are used widely to provide energy for cooking, heating, and lighting. Women and young children experience high levels of smoke exposure when meals are cooked over open fires due to partial combustion of fuel and poor ventilation.^5^^,^^6^ Household air pollution from open fires is a major threat to health, ranking 10th in the World Health Organization (WHO) comparative risk assessment for the global burden of disease.^7^ The 2017 Global Burden of Disease Study suggests there are 1.6 million deaths attributable to household air pollution annually, of which approximately one-half of a million are deaths from pneumonia in young children.^8^ In Malawi, where at least 95% of households depend on biomass as their main source of fuel and where household air pollution levels are high, biomass smoke exposure has been thought to be responsible for a substantial burden of this disease.^5^^,^^6^^,^^9^

In this context, we did a cluster-randomized controlled trial of introducing cleaner-burning biomass-fueled cookstoves to prevent pneumonia in children <5 years od in rural Malawi (the Cooking and Pneumonia Study [CAPS]).^10^ CAPS included 10,750 children from 8626 households across 150 community-level clusters with 10,543 children from 8470 households contributing 15,991 child-years of follow-up data to the intention-to-treat analysis. Although the Integrated Management of Childhood Illness (IMCI)-defined pneumonia incidence rate overall was substantial (15.7 per 100 child-years), we found no difference in the pneumonia incidence rate between the intervention and control groups (incidence rate ratio, 1.01; 95% CI, 0.91 to 1.13; *P* = .80). To explore possible explanations for this finding, we now report data from CAPS on (1) the association between exposure to carbon monoxide (CO) and carboxyhemoglobin (COHb) and pneumonia, (2) a comparison of CO exposures and COHb levels in children with and without an episode of pneumonia during the trial, and (3) the effects of the intervention on personal exposure to CO and COHb levels among the one-in-four children who underwent these measurements. The primary CAPS trial outcome data and CO and COHb data collected at the point of recruitment to CAPS have been published previously.^10^^,^^11^

## Methods

### Study Design

CAPS was a cluster-randomized controlled trial with two arms of equal size that compared the effects of a cleaner-burning biomass-fueled cookstove intervention to the continued use of traditional open fire cooking on pneumonia incidence (primary outcome previously reported) and CO exposures (secondary outcomes) in children <5 years old living in rural Malawi over a 2-year period.

### Setting

We defined 150 community-level clusters within villages across two districts of Malawi; Chikwawa in the southern Shire river valley and Karonga on the northern Malawi lakeshore. The Malawi College of Medicine Research Ethics Committee (ethics committee reference number P.11/12/1308) and the Liverpool School of Tropical Medicine Research Ethics Committee (ethics committee reference number 12.40) approved the CAPS trial protocol. Study registration ISRCTN 59448623.

### Participants

After community engagement exercises with village leaders and communities and the identification of a representative for each cluster, households with at least one child up to 4.5 years old were invited to participate. Written informed consent (or witnessed thumbprint for those unable to read and write) was obtained at cluster and household-level (parent or guardian of child) prior to participation. The trial was open to all consenting households with a child in the eligible age range. Households that became eligible for inclusion during the course of the trial (through birth, adoption, or in-migration) were recruited up to 6 months before the end of the trial.

### Randomization and Masking

Clusters were allocated to intervention and control arms with the use of a computer-generated randomization schedule with stratification by site, distance from health center, and cluster size. An additional level of randomization was done to select participants for this study with the use of a randomization function built in to the electronic case report form that selected one in four children who were included in CAPS to be invited to participate in the substudy. Individuals were assigned to the randomization arm based on their cluster membership at baseline.

### Procedures

Intervention households received two cleaner-burning biomass-fueled cookstoves (Philips HD4012LS), a solar panel, and user training. A fan incorporated into these cookstoves improves combustion efficiency; smoke emissions have been found to be reduced by approximately 90% compared with the open fire in laboratory testing. Cookstoves were repaired and replaced as needed. Control households continued using traditional cooking methods (typically open fires). At the start of the trial, control households were informed that they would receive the intervention at the end of the study period for equity and to maximize retention. Each household was visited every 3 months by fieldworkers; although by the time the 21-month visit was due, we were 3 months behind schedule and so moved directly onto the final 24-month visit.

### Primary Outcome

The primary outcome was the incidence of WHO IMCI-defined pneumonia diagnosed by physicians, medical officers, or other appropriately trained staff at local healthcare facilities that were accessed routinely by trial participants who were unaware of intervention allocation. Secondary outcomes included severe IMCI-defined pneumonia and severe pneumonia with oxygen saturation <90%.

### Carbon Monoxide Exposure Outcomes

We measured personal exposure to CO directly in all participating children with EasyLog-USB-CO Lascar monitors (Lascar Electronics) that measured CO with an electrochemical cell and indirectly in children aged ≥6 months with the use of Masimo Radical-57 Rainbow SET Pulse CO-Oximeters (Masimo Corporation) that measured COHb levels transcutaneously using a pediatric sensor that was placed on a digit.^11^ The CO-Oximeters were checked daily with the manufacturer’s testing device (Masimo Rainbow Tester) to quality assure the measurements. Because the finger sensors for the COHb levels were suitable only for children above the age of 6 months (according to to the manufacturer’s instructions), children below this age had personal exposure to CO but not COHb levels measured. CO and COHB levels were measured both at baseline and at 6-month follow-up visits.

CO monitors were set to take measurements every 30 seconds and then placed in a fabric holder that was worn around the neck of the child so that the monitor was close to the child’s breathing zone. CO monitors were worn continuously for 48 hours, except during sleeping hours when they were placed beside the child. At the end of the monitoring period, a fieldworker visited the child’s home to retrieve the monitor and upload the data onto a study laptop. At this same visit, a short questionnaire about factors potentially related to CO exposure was completed, and COHb levels were measured by taking three recordings (four when there was a >6% variation between recordings).

Data from CO monitors were downloaded onto a study laptop in the field at the time they were collected; the questionnaire and COHb data were entered into electronics that had been programmed onto smart phones (Samsung Galaxy S3) with the use of Open Data Kit software. All data were transferred to a secure server at each study site when the fieldworkers returned to base, whereupon data checking and cleaning were done before being forwarded on to a central secure server in Liverpool, UK, ready for analysis.

### Sample Size

Sample size considerations for CAPS have been reported previously. In brief, we aimed to include 150 community-level clusters that represented approximately 10,600 eligible children to provide, over the 2-year study period, approximately 21,200 child-years of follow-up data and 90% power to detect a 20% difference in the pneumonia incidence rate between the intervention and control groups, assuming a baseline rate of 5 per 100 child years. One in four children included in CAPS were assigned randomly for inclusion in this substudy.

### Statistical Methods

Individual characteristics at baseline were summarized by frequency (proportion) or median (interquartile range [IQR]), as appropriate. Incidence (95% CI) of pneumonia was estimated by taking the total number of pneumonia events over total sum of person-time (randomization until last contact) with Poisson exact CIs. R software was used to process the CO monitor measurements. CO monitor files with <20 hours of measurement (2 files) were excluded. Measurements exceeding the upper limit of detection (LOD) of the instrument were set to the upper limit (1000 parts per million (ppm), and the total minutes above LOD per 24 hour-period calculated. Any files with >14 minutes of observation above the upper limit were excluded (0 files). Each period of monitoring over 20 hours was split into one or two periods, the first from 0 to 24 hours, the second from 24 to 48 hours, labelled period “A” and “B.” If period B was <20 hours in duration, only the first period was retained. Four files with incorrect data based on graphs and outlying values (malfunctioning monitors) were removed. Values below the LOD were set to 0.5∗LOD. CO measurements for each individual 24-hour period were summarized with the use of arithmetic mean, maximum, geometric mean, and geometric SD, which resulted in a single value for each 24-hour monitoring period. We also calculated the minutes above and below the instrument LOD. These values were then summarized as median (IQR). Mean CO was transformed to log_10_ for modeling purposes, although it is described in text and tables as parts per million. Summary statistics were calculated, and group differences (95% CIs) were estimated. Group comparisons were by two-sided Wilcoxon rank sum test.

Generalized estimating equations with the use of a Poisson model with log link function were used to estimate associations between number of episodes of pneumonia (alternatively severe pneumonia or severe pneumonia with oxygen saturation <90%) and exposure measures, fitting separate models to assess CO exposure and COHb measures, assuming an exchangeable correlation structure, adjusting for age, sex, presence of smokers in the household, visit, and randomization arm, and including an offset for duration of follow up. Clustering was on time periods (due to possibility of two 24-hour monitoring periods in the same visit) within individuals within village, and CO was entered into these models as log_10_ CO. Age-stratified models were run for CO exposure, considering visits up to or after 6 months of age separately. Results are presented as estimated incident rate ratios (IRR) and 95% CIs. Multilevel linear mixed models were fit with the use of CO measures as a time-varying outcome to estimate adjusted association with the randomization arm. Separate models were run with COHb and log_10_ CO as the outcome variable. Age, sex, visit, and the presence of smokers in the household were included as adjustment factors, and random intercepts were fit for individuals, nesting time period (due to possibility of two 24-hour monitoring periods), and for cluster at randomization.

## Results

### Participants

**Figure 1:**
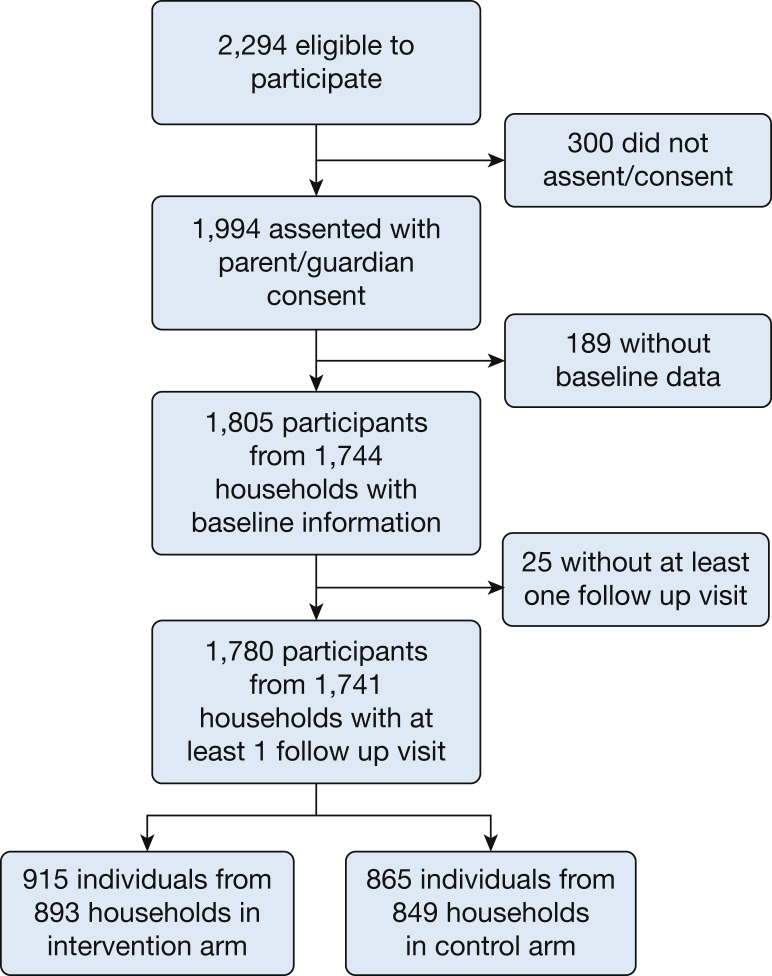
Consort diagram.

Between December 9, 2013, and February 28, 2016, 2294 children were invited to take part in this study, of whom 1994 assented with parental/guardian consent, and 1805 from 1744 households contributed data at baseline. We lost 25 children (13 intervention; 12 control) from 3 households between the baseline and first follow-up visit. Data were therefore available from 1805 children (928 intervention; 877 control) at baseline and from 1780 children (915 intervention; 865 control) with at least one follow-up visit who were included in the dataset for analysis. The mean age of participating children was 25.6 (SD, 15.5) months; 50.6% were female. Participants’ characteristics at baseline were similar in the intervention and control groups (Table 1). The last participant follow-up visit was on September 14, 2016 (Fig 1).

**Table 1:** Baseline Characteristics of the Intention-to-Treat Population

Variable	Intervention (n = 928)	Control Subjects (n = 877)
Individual-level data		
Age, mean [SD], mo	25.7 [15.8]	25.5 [15.2]
Female, No. (%)	454 (48.9)	460 (52.4)
Vaccination status (course completed),^a^ No. (%)		
Diphtheria, tetanus, pertussis, hepatitis B, and *Haemophilus influenzae* B	522 (84.1)	534 (87.3)
Pneumococcal conjugate	378 (60.9)	391 (63.9)
Polio	496 (79.9)	517 (84.5)
Rotavirus	298 (48.0)	291 (47.5)
Measles	391 (63.0)	411 (67.2)
Had pneumonia at least once in the preceding 12 mo, No. (%)	146 (15.7)	161 (18.4)
Had a cooking-related burn in the preceding 3 mo, No. (%)	50 (5.4)	65 (7.4)
Household-level data	N = 892	N = 851
Fuel used regularly for cooking,^b^ No. (%)		
Electricity	0 (0)	0 (0)
Gas	0 (0)	0 (0)
Paraffin/kerosene	0 (0)	2 (0)
Charcoal	107 (12.0)	156 (18.3)
Wood	489 (54.8)	441 (51.8)
Crop residues	304 (34.1)	278 (32.7)
Dung	8 (0.1)	2 (0)
Other	1 (0)	0 (0)
Tobacco smoker in the household, No. (%)	146 (16.4)	126 (14.8)
Daily or almost daily exposure to smoke,^b^ No. (%)		
Burning rubbish	362 (40.6)	308 (36.2)
Cooking as business	129 (14.5)	113 (13.3)
Paraffin/kerosene lamps	24 (2.7)	18 (2.1)
Beer production	14 (1.6)	4 (0)
Mosquito coils	9 (1.0)	17(2.0)
Brick production	33 (3.7)	43 (5.1)
Other sources	17 (1.9)	6 (0.7)
Source of drinking water,^b^ No. (%)		
Tap to house	74 (8.3)	77 (9.1)
Shared communal tap	99 (11.1)	69 (8.1)
Covered well	52 (5.8)	53 (6.2)
Open well	47 (5.3)	56 (6.7)
Bore hole	376 (42.2)	358 (42.1)
Lake or river	62 (7.0)	30 (3.5)
Other	0 (0)	1 (0)
Toilet facilities, No. (%)		
Water toilet	6 (0.7)	2 (0.2)
Ventilated improved pit	1 (0)	4 (0.5)
Simple pit latrine	703 (78.8)	698 (82.0)
None	182 (20.4)	147 (17.3)
Experienced a time in the last year when there was not enough food for the household to have its normal meals, No. (%)	491 (55.0)	465 (54.6)
Experienced a time in the last year when the household did not have money to buy bathing soap, No. (%)	603 (67.6)	574 (67.5)

a Vaccination status available for 1233 children (621 intervention; 612 control subjects).

b A household could give multiple responses.

### CO and COHb Measurements

CO and COHb measurements were done at baseline on 1697 (900 intervention; 837 control) and 1574 (807 intervention; 767 control) children, respectively. The median of 24-hour averaged CO exposures at baseline was 0.45 ppm (IQR, 0.18 to 0.92 ppm) and the mean of COHb levels was 5.85% (SD, 3.36%); similar in intervention and control groups (Table 2). Over the 2 years of follow-up, there were 5521 (2911 intervention; 2620 control) and 4065 (2113 intervention; 1952 control) measurements of 24-hour CO exposure and COHb levels, respectively. A total of 377 24-hour CO periods had all measurements below the LOD (204 intervention, 173 control).

**Table 2:** Summary Statistics of Carbon Monoxide and Carboxyhemoglobin Over Visit Schedule

Variable	Baseline	Follow-up Visit			
		1	2	3	4
Mean CO, median (IQR), ppm	0.45 (0.18 to 0.92)	0.27 (0.13 to 0.84)	0.25 (0.10 to 0.88)	0.25 (0.09 to 0.88)	0.25 (0.09 to 0.83)
Maximum CO, median (IQR), ppm	32.8 (19.0 to 54.0)	26.5 (12.0 to 52.0)	24.0 (8.5 to 58.0)	20.5 (7.0 to 49.0)	22.5 (8.5 to 40.5)
Control arm: mean CO, median (IQR), ppm	0.49 (0.21 to 1.02)	0.33 (0.15 to 0.89)	0.25 (0.09 to 1.08)	0.25 (0.09 to 0.76)	0.65 (0.22 to 0.88)
Intervention arm: mean CO, median (IQR), ppm	0.48 (0.18 to 0.86)	0.25 (0.12 to 0.79)	0.25 (0.12 to 0.79)	0.26 (0.10 to 0.92)	0.25 (0.08 to 0.50)
Difference (intervention to control subject) in mean CO, ppm (95% CI)	0.37 (−0.17 to 0.93)	1.09 (−0.16 to 2.34)	−0.36 (−1.07 to 0.33)	−0.09(−1.35 to 1.17)	−0.05 (−0.93 to 0.86)
Average minutes of observation (per 24-h period), No.	1407	1419	1420	1427	1437
COHb, mean (SD), %	5.85 (3.36)	5.33 (3.73)	5.26 (3.65)	5.40 (3.44)	5.23 (3.54)
Control arm: COHb, mean (SD), %	5.94 (3.73)	5.34 (3.73)	5.48 (3.64)	5.68 (3.44)	5.69 (3.63)
Intervention arm: COHb, mean (SD), %	5.75 (3.74)	5.31 (3.74)	5.05 (3.65)	5.13 (3.43)	4.88 (3.44)
Difference (intervention to control subject) in COHb (95% CI)	−0.18 (−0.52 to 0.15)	−0.03 (−0.41 to 0.34)	−0.42 (−0.83 to −0.02)	−0.55 (−1.01 to −0.09)	−0.81 (−1.56 to −0.06)

CO = carbon monoxide; COHb = carboxyhemoglobin; IQR = interquartile range; ppm = parts per million.

### Exposure-Response Analysis

**Figure 2:**
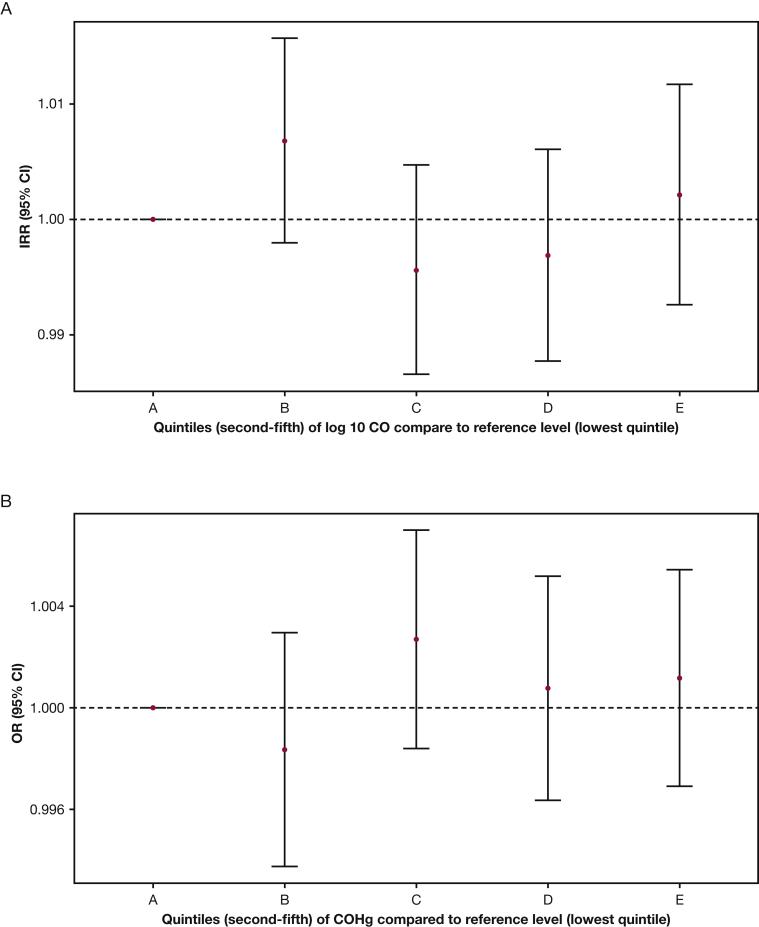
A-B, Estimated incident rate ratios (95% CI) for the association between pneumonia and log10 (parts per million) (A) and pneumonia and carboxyhemoglobin exposure exposure fit as a categoric variable in quintiles (B). The lowest quintile (lowest 20% of exposure values) is the reference level for other estimates. Models are adjusted for age, presence of smokers in the household, sex, randomization arm, visit number, and follow-up time, and are clustered on an individual period. CO = carbon monoxide; COHb = carboxyhemoglobin; IRR = incident rate ratio.

There were 517 pneumonia episodes that provided an incidence rate of 0.176 (95% CI, 0.161 to 0.191) per person-year. Adjusted generalized estimating equation models demonstrated no evidence of association between CO (IRR, 1.0; 95% CI, 0.967 to 1.014; *P* = .53) or COHb (IRR, 1.00; 95% CI, 0.993 to 1.003; *P* = .41) and the rate of pneumonia. Analysis of secondary endpoints was similar with no association identified for severe pneumonia (n = 192 episodes) and CO (IRR, 0.95; 95% CI, 0.90 to 1.01; *P* = .083) or COHb (IRR, 1.01; 95% CI, 0.998 to 1.02; *P* = .11) nor for severe pneumonia with oxygen saturation <90% (n = 27 episodes) and CO (IRR, 1.03; 95% CI, 0.92 to 1.15; *P* = .67). Severe pneumonia with oxygen saturation <90% was associated with COHb (IRR, 0.95; 95% CI, 0.91 to 0.99; *P* = .011), where higher COHb levels were mildly protective. However, once corrected for false-discovery rate, no results remain statistically significant at a probability value of <.05. Each model was adjusted for age, presence of smokers in the household, sex, randomization arm, visit number, and follow-up time and was clustered on an individual period. Age stratified subanalyses were run, considering episodes occurring up to and including 6 months of age separately from those occurring after 6 months of age. Results were unchanged with all IRR estimates for CO association (where appropriate) between 0.95 and 1.06, and none were statistically significant (Fig 2).

### CO and COHb Levels in Children With and Without a Pneumonia Outcome

Median CO exposures in children with and without an episode of pneumonia during the trial were 0.31 ppm (IQR, 0.13 to 0.92 ppm) and 0.36 ppm (0.15, 0.88 ppm), respectively, giving a difference (pneumonia compared with no pneumonia) of 0.02 (95% CI, −0.58 to 0.62; *P* = .18). Children who experienced an episode of severe pneumonia had median CO of 0.44 ppm (IQR, 0.16 to 1.23 ppm), and those with severe pneumonia with low oxygen saturation had a median CO of 0.25 ppm (IQR, 0.20 to 0.57 ppm). Mean COHb levels in children with and without an episode of pneumonia during the trial were 5.34% (SD, 3.54%) and 5.46% (SD, 3.64%), respectively, giving a difference of −0.09 (95% CI, −0.33 to 0.16; *P* = .49). Children with an episode of severe pneumonia had mean COHb of 5.24% (SD, 3.99%), and those with an episode that included low oxygen saturation had mean COHb of 4.03% (SD, 2.27%).

### Intention-to-Treat Analysis

**Figure 3:**
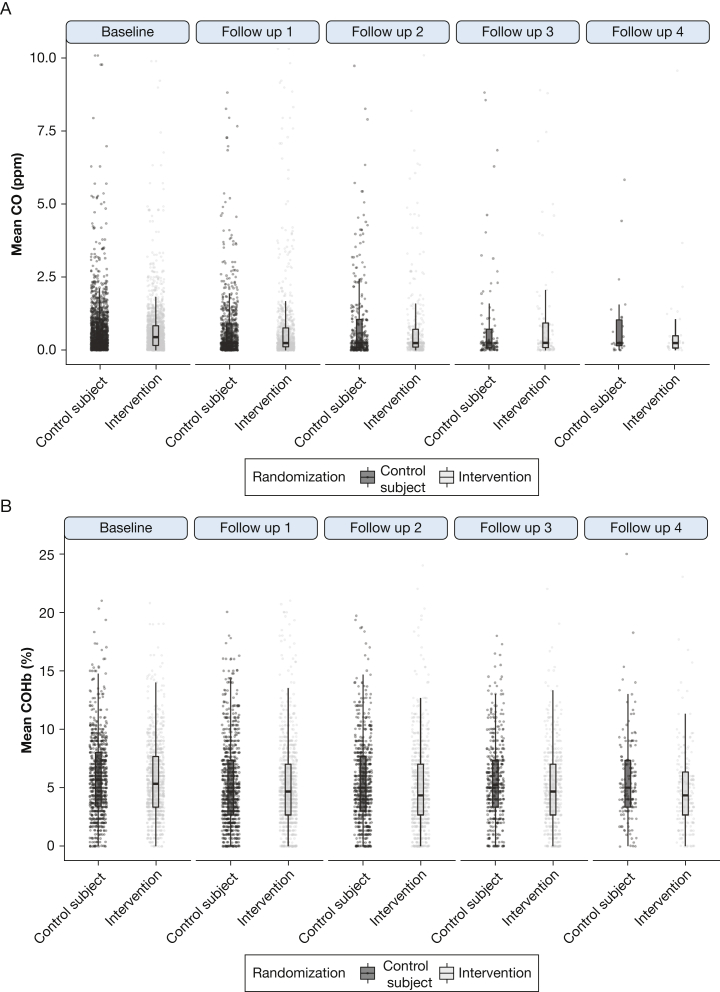
A, Mean carbon monoxide (parts per million) measured at baseline (visit 1) and the following four follow-up visits (visits 2 to 5) for each individual. The plotted shape represents a density estimate. The Y-axis truncated at a value of 10 (maximum observed mean carbon monoxide = 303 parts per million). B, Mean carboxyhemoglobin per individual measured at at baseline and the following four follow-up visits for each individual. The plotted shape represents a density estimate. The Y-axis truncated at a value of 25 (maximum observed carboxyhemoglobin = 26). ppm = parts per million. See Figure 2 legend for the expansion of the other abbreviations.

Median exposure to CO in the intervention and control groups was 0.34 ppm (IQR, 0.15 to 0.81) and 0.37 ppm (IQR, 0.15 to 0.97), respectively, giving a difference of −0.46 ppm (95% CI, −0.95 to 0.012; *P* = .06) (Fig 3A). In linear mixed models, after adjustment for time, age, sex, and the presence of smokers in the household, the difference was −0.029 ppm (95% CI, −0.12 to 0.06). Taking the intervention group alone, median CO was 0.50 ppm (IQR, 0.02 to 0.80 ppm) and 0.2 ppm (IQR, 0.1 to 0.8 ppm) before and after the intervention was received, respectively (*P* < .08).

Mean COHb levels in the intervention and control groups were 5.31% (95% CI, 5.17 to 5.43) and 5.60% (95% CI, 5.47 to 5.74), respectively, giving a difference of −0.30% (95% CI, −0.48 to −0.11; *P* = .0017) (Fig. 3B). After adjustment in linear mixed models for time, age, sex, and the presence of smokers in the household, the difference was −0.24% (95% CI, −0.57 to 0.07). Taking the intervention group alone, mean COHb levels were 5.75% (SD, 3.27) and 5.13% (SD, 3.53) before and after the intervention was received, respectively (*P* < .0001).

## Discussion

This longitudinal study of personal exposure to household air pollution (assessed through measurement of CO and COHb) and its association with pneumonia in children under the age of 5 years in Malawi included 1780 children who contributed 3549 and 4065 measurements of CO exposure and COHb levels, respectively, over 2 years of follow up. Although personal exposure to CO was low, COHb levels were elevated consistently over the 2 years of follow up. We found no association between exposure to CO averaged over a 24-hour period or levels of COHb and the incidence of pneumonia. In addition there were no differences in the average levels of CO exposure or COHb percentage in children with or without an episode of pneumonia during the trial. There was no difference observed in average CO exposure, and there was only a minor difference in COHb levels between intervention and control group participants, which is consistent with the lack of intervention effect that was observed in the main intention-to-treat analysis.

In our main clinical trial report, we put forward two main explanations for the lack of effect of the intervention on pneumonia incidence: (1) potential effects of the intervention may have been overwhelmed by other sources of air pollution, and (2) the intervention did not reduce exposures sufficiently. An alternative explanation is that the causal relationship between exposure to household air pollution and pneumonia in children is not as strong as previously thought and that confounding, for example by the many dimensions of poverty, is also a factor. If true, then an intervention that aimed to reduce exposure to household air pollution from cooking in isolation would have less potential to impact on this outcome.

The low (below WHO 24-hour CO exposure level of 7 ppm^12^) personal CO exposure levels seen over the course of the trial in both trial arms is consistent with other studies of household air pollution performed in Africa and elsewhere (eg, in Randomised Exposure Study of Pollution Indoors and Respiratory Effects [RESPIRE] it was 3.4 ppm).^13^) This could be related to lower levels of this pollutant in combusted biomass fuel relative to other pollutants and/or where measurements are conducted when cooking is outside the home.^14^, ^15^, ^16^ It also suggests that the measurement of personal CO exposure for short periods of time may not always be a sensitive indicator for studies of the effects of household air pollution exposure reduction interventions, especially when cooking is done outdoors, and there is no straightforward way to confirm that these personal monitoring devices are worn as instructed. The possibility of poor concordance with wearing the devices is another potential explanation for the apparently low CO exposures. In contrast, COHb levels were high and inconsistent with the personal CO exposure data. This finding might suggest that exposure to CO as a component of household air pollution is high after all and that COHb may offer a more sensitive and more biologically relevant indicator of CO exposure. The finding of a clearer, albeit weak, signal of effect of the intervention on COHb than CO is consistent with this conclusion. We acknowledge, however, that our interpretation of COHb data is limited in the very youngest children in whom pneumonia incidence is highest because of limitations of the technology we were using meant that we were unable to measure COHb in children under the age of 6 months.

It is also possible that there was a behavior change while the children were wearing the monitors that led to lower exposures. At the same time, it is PM_2.5_ rather than CO exposure that has been considered to be implicated mechanistically in increasing the risk of pneumonia by way of impairing host defenses. However, the work that we have done in Malawi (at the same time as the work described in this article) and others have done elsewhere in the world suggests that these two exposures are not always well correlated (*R*^2^ = 0.11) in our CAPS-linked study of noncommunicable respiratory disease and air pollution exposure in Malawian adults^17^; therefore, CO exposure cannot be assumed to be an accurate proxy for PM_2.5_ exposure, especially where cooking is done outside.^17^^,^^18^ In general, PM_2.5_ is more complex and costly to measure than CO and requires equipment that is not well-suited for personal monitoring in young children or on the kind of scale we achieved in this study. We are left therefore with uncertainty about the extent to which children in rural Malawi are exposed to household air pollution that would have been measured by PM_2.5_ had this been feasible, about the clinical relevance of reductions in these exposures of the magnitude we observed, and about the value of measuring CO as an indicator of household air pollution exposure in studies such as this. In more densely populated urban settings where biomass use is common and there are multiple other sources of air pollution, this may not be the case.

Although there is biologic plausibility for a causal link between exposure to household air pollution and pneumonia in young children and strong indirect evidence, there is a relative paucity of direct evidence for this.^4^, ^5^, ^6^^,^^19^^,^^20^ Many of the individual exposure-response studies to date have been limited by indirect assessments of either exposure or outcome while pooled/metaanalyses that have been used to create exposure-response curves have drawn substantially on extrapolation from studies of ambient and tobacco-related air pollution exposures.^5^^,^^19^^,^^20^ An exception is the RESPIRE trial of a chimney cookstove in Guatemala where an exposure-response relationship between measured CO (as a modeled proxy for PM_2.5_) and pneumonia during the first 18 months of life was observed.^21^ Unlike many of the published studies of household air pollution and childhood pneumonia, cooking was done indoors by RESPIRE households, and the infant typically was carried on the mother’s back during cooking periods. A recent systematic review of household air pollution exposures and pneumonia in children found that, although associations were seen when questionnaires were used for exposure assessment, these associations were not usually seen (RESPIRE was an exception) when air pollutants that included CO and particulate matter were measured directly.^19^ This conclusion is also consistent with the findings of our other recently published work from Malawi that has found no evidence that exposure to household air pollution was associated with pneumonia in adults, with respiratory symptoms or lung function in children and adults, or with the rate of decline on lung function in adults.^17^^,^^22^, ^23^, ^24^, ^25^

The strengths of this study include that it is the largest longitudinal study of personal exposure to CO and COHb levels in children in rural Africa and that, as part of CAPS, it benefited from the randomized controlled trial design, conduct, completeness of quality-assured data collection, and analysis. Lost to follow-up and use of an aggregate endpoint (total pneumonia episodes) may impact the accuracy of estimated associations, but the main limitation is the lack of tools to measure personal exposure to PM_2.5_ in young children in a straightforward and cost-effective way that can be done frequently for prolonged durations of time on large numbers of participants. Although CO monitors are available that can meet these requirements, CO exposure is less biologically relevant to pneumonia development and may be a poor surrogate marker of PM_2.5_ exposure, especially in settings where cooking is done outdoors.

We found that young children in rural Malawi experience exposure to household and other types of air pollution on a day-to-day basis when questionnaire data are considered but that data from more direct measurements (personal CO exposure and COHb levels) are contradictory. We found no association between exposure to CO and pneumonia incidence and no effect of the CAPS intervention on these exposures, which suggests that CO may not be an appropriate measure of household air pollution exposure in settings like rural Malawi and that there is a need to develop ways to measure particulate matter exposures directly in young children instead. There is also a need to reexamine the role of cleaner-burning cookstoves and fuels as stand-alone health interventions. Addressing individual sources of air pollution alone is unlikely to be sufficient for improving health; instead, a comprehensive approach to emission control from all sources is required to improve air quality both inside and outside the home.
